# Frequency of superoxide dismutase 1 c.118: G>A mutation associated with canine degenerative myelopathy in German Shepherd dogs from Uruguay and Paraguay

**DOI:** 10.14202/vetworld.2024.2992-2997

**Published:** 2024-12-30

**Authors:** Rody Artigas, Carolina Menchaca, Liz Castro, Alejandra Mondino, Yamila Perdomo, Facundo Bera, Sofía Stagno, Micaela Borca, Natalia Mendez, José Ramirez, Silvia Llambí

**Affiliations:** 1 Unidad Académica de Genética y Mejora Animal. Departamento de Producción Animal y Salud de los Sistemas Productivos. Facultad de Veterinaria, Udelar, Uruguay; 2 Departamento de Genética. Facultad de Veterinaria, UNA, Uruguay; 3 Department of Clinical Sciences, North Carolina State University, Raleigh, North Carolina, USA

**Keywords:** degenerative myelopathy, genetic disease, German Shepherd dog, superoxide dismutase 1 gene

## Abstract

**Background and Aim:**

Canine degenerative myelopathy (DM) is an autosomal recessive inherited disease that affects different dog breeds. It has an invariably fatal outcome once the clinical symptoms begin. This study aimed to investigate the population behavior of the mutation superoxide dismutase 1 (SOD1) c.118: G>A responsible for the high risk of developing DM in two populations of German Shepherd dogs from Uruguay and Paraguay.

**Materials and Methods:**

A total of 158 German Shepherd dogs from Uruguay (n = 114) and Paraguay (n = 44) were analyzed. Genomic DNA was extracted from peripheral whole blood. The SOD1 c.118: G>A mutation was identified by polymerase chain reaction-restriction fragment length polymorphism and subsequently validated using sequencing. Allelic and genotypic frequencies and Hardy–Weinberg equilibrium were calculated for both populations. The rate of clinical progression was evaluated in animals homozygous for the mutation.

**Results:**

The frequencies of allele A associated with a higher risk of DM, were 0.15 and 0.23 in Paraguay and Uruguay, respectively. Paraguay’s population was found to be in Hardy–Weinberg equilibrium (p = 1.00), whereas the population of dogs from Uruguay deviated from equilibrium (p = 0.008). When comparing the populations, no significant difference was observed in the distribution of genotypes (p = 0.26). When evaluating the clinical progression rate, all animals aged >10 years showed clinical symptoms compatible with DM.

**Conclusion:**

This study demonstrated for the first time the presence of the SOD1:c118 G>A mutation in German Shepherd dogs from Uruguay and Paraguay. The frequency detected in Uruguay was significant. Although the frequency was lower in Paraguay, the allele was present. This demonstrates the need to implement genotyping tests as part of a possible DM control program in both countries studied.

## Introduction

Canine degenerative myelopathy (DM) (OMIA 000263-9615) is a progressive, autosomal recessive hereditary disease affecting the spinal cord. It was initially detected in a German shepherd dog. However, currently, the responsible mutation has been detected in other breeds, such as Pembroke Welsh Corgi, boxer, Rhodesian ridgeback, Chesapeake Bay retriever, Bernese mountain dog, miniature and standard poodle, pug, Siberian husky, Cavalier King Charles spaniel, American Eskimo, Cardigan Welsh corgi, golden retriever, Kerry blue terrier, soft coated wheaten terrier, wirehaired fox terrier, Hovawart, among others [1].

The onset of signs is highly variable between breeds, although it usually begins at 5 years of age, with an average of 9 years in large breeds [2]. The neurodegenerative process begins in the thoracolumbar region of the spinal cord (T3-L3) and progresses to the cranial and caudal areas of the spinal cord [3].

The earliest clinical symptoms include proprioceptive ataxia and mild spastic paralysis of the pelvic limbs, which progresses to paraplegia, loss of sphincter control, and tetraplegia at later stages [2, 4, 5]. The clinical course of the disease is rapid, with non-ambulatory paraparesis observed in large breed dogs 6–9 months after symptoms begin, at which time owners opt for euthanasia [2]. In small-breed dogs, assistance can be given longer, thereby increasing their survival [4, 6].

The gene responsible for this disease is *SOD1*, which encodes the enzyme superoxide dismutase 1 (SOD1) [7]. This enzyme plays a fundamental role in the oxidative homeostasis of the central nervous system by converting superoxide radicals to hydrogen peroxide and oxygen [8]. To date, two mutations in *SOD1* gene have been identified as responsible for the phenotype. The first of them is a G>A transition (c.118G>A) in the second exon, which produces a change from a glutamine to a lysine at residue 40 (E40K) of the protein [7]. This mutation is considered ancestral because it is common to different breeds, including crossbred dogs [9]. The second mutation, exclusive to the Bernese Mountain Dog breed, is located in exon 1 and consists of an A>T transversion (c.52A>T) that leads to a change from threonine to serine in residue 18 (T18S) of the protein [10].

The definitive diagnosis of DM is made only by postmortem through histopathological studies. In the spinal cords of affected animals, insoluble aggregates of SOD1 are observed in neuronal soma, and these aggregates can be detected by immunohistochemistry [7, 9, 11]. In this situation, genotyping of symptomatic animals is particularly important for diagnosis.

The population distribution of the c.118G>A mutation has been analyzed in different breeds and populations worldwide. Epidemiological surveys must be conducted in each country and region to establish prevention schemes [12].

This study aimed to determine the behavior of the c.118G>A mutation associated with DM in population samples of German Shepherd dogs from two countries in South America, Uruguay and Paraguay.

## Materials and Methods

### Ethical approval and Informed consent

This study was approved by the Commission on Ethics in the Use of Animals (CEUA) (approval no. 1278). Samples were obtained through written informed consent from the owners.

### Study period and location

The study was conducted from March 2021 to March 2024 at the Facultad de Veterinaria-Udelar, Montevideo-Uruguay.

### Animals

Peripheral whole blood samples with EDTA were collected from German shepherd dogs from Uruguay (n = 114). The animals were of both sexes, of different ages, belonging to different regions of the country, and were unrelated. Samples from Paraguay (n = 44) belonged to the DNA bank of the Department of Genetics and Zootechnics at the Faculty of Veterinary Sciences of the National University of Asunción and came from animals from different regions of the city of Asunción. The animals used belonged to both sexes, were of different ages, and were unrelated. At the time of sampling, the owners reported that the animals were clinically healthy.

### DNA extraction

Genomic DNA extraction was performed from 200 μL of blood using a commercial Genomic DNA Purification Kit (Fermentas, USA) according to the manufacturer’s instructions. The isolated DNA was quantified for concentration and purity using a DeNovix DS11 drop spectrophotometer (DeNovix Inc., USA). The samples were standardized to a final concentration of 50 ng/μL of DNA.

### Genotyping of the c.118G>A mutation in exon 2 of *SOD1* gene

Genotyping of the c.118G>A mutation in exon 2 of the *SOD1* gene was performed using polymerase chain reaction (PCR)-restriction fragment length polymorphism (RFLP) techniques. A 292-bp fragment of *SOD1* gene amplified by PCR using the primers F: 5′-AGTGGGCCTGTTGTGGTATC-3′ and R: 5′-TCTTCCCTTTCCTTTCCACA-3′, which were previously designed by Holder *et al*. [13]. Amplification was performed using a Multigene II thermocycler (Labnet Inc. Global, USA) in a final reaction volume of 25 μL containing 50 ng of genomic DNA, 1 μL of each primer (10 pmol/μL), 12.5 μL of ImmoMix (Bioline, Australia), and Miliq water to complete the volume. The amplification program consisted of 95°C for 10 min, followed by 35 cycles of 94°C for 40 s (denaturation), 55°C for 30 s (annealing), and 72°C for 1 min (extension), with a final extension at 72°C for 10 min. Amplicons were detected by electrophoresis in 1.5% agarose gel using GoodViewTM (SBS Genetech Co., Ltd., China) as a stain.

The amplicons were digested in a reaction containing 2.5 μL of digestion buffer (NEB, New England Biolabs, Massachusetts, USA), 11.5 μL ultrapure water, 1 μL Acu I enzyme (NEB, New England Biolabs), and 10 μL of PCR product, resulting in a final volume of 25 μL. The reaction was incubated overnight at 37°C, followed by inactivation of the enzyme at 65°C for 20 min. To determine the genotype of the animals, digestion products were evaluated by electrophoresis in 1.5% agarose gels stained with GoodViewTM (SBS Genetech Co., Ltd).

### RFLP validation

Three animals of each genotype (GG, GA, AA) were sent to Macrogen (Korea) for sequencing in both directions to validate the RFLP. Sequence analysis was performed using the open-access program BioEdit 7.2.5 (http://www.mbio.ncsu.edu/BioEdit/bioedit.html).

### Population analysis

Allele and genotypic frequencies were calculated by direct counting. Hardy–Weinberg equilibrium was calculated using Fisher’s exact test. All calculations were performed using the free license program Genepop 4.2 (available at: https://genepop.curtin.edu.au/).

### Clinical progression rate

The rate of clinical progress was calculated according to the number of dogs with a risk genotype presenting symptoms suggestive of DM, expressed as a percentage, as established by Maki *et al*. [14]. For this purpose, a neurological examination of the Uruguayan dogs was performed. The data were analyzed in conjunction with previously reported data by Maki *et al*. [14].

## Results

### Allele frequency

**Figure-1:**
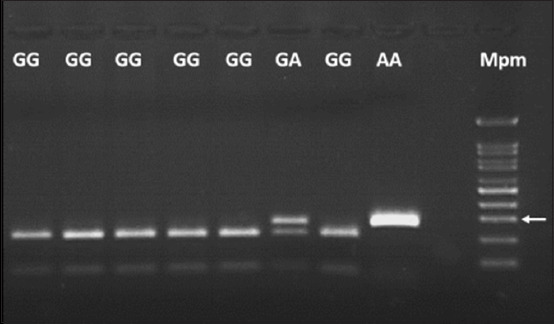
1.5% agarose gel electrophoresis of digestion patterns with the *AcuI enzyme* at the superoxide dismutase 1:c118 G>A site. GG=Homozygous dominant genotype, GA=Heterozygous genotype, AA=Homozygous recessive genotype related to an increased risk of degenerative myelopathy. Mpm=100 bp molecular weight marker. The white arrow indicates the 300-bp Mpm line.

**Figure-2:**
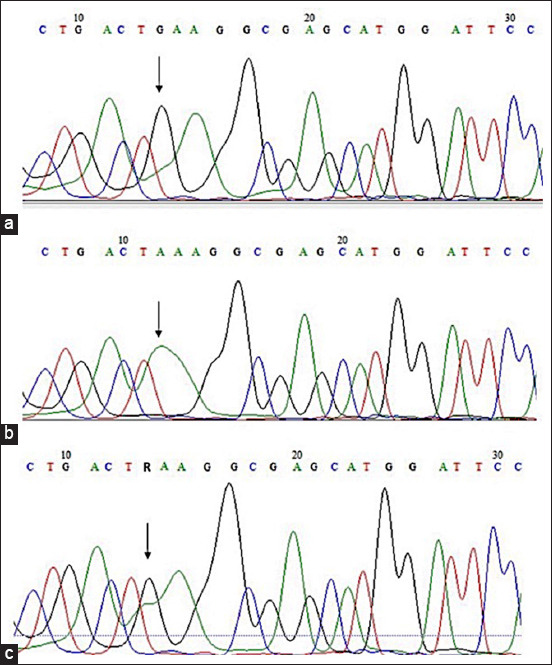
Electropherograms of amplicons of superoxide dismutase 1 gene containing the c118 G>A mutation site. (a) Homozygous dominant genotype GG. (b) Homozygous recessive AA genotype associated with an increased risk of degenerative myelopathy. (c) Heterozygous GA genotype.

The amplified fragment of *SOD1* gene showed an amplicon of 292 bp. After digestion with the *AcuI* enzyme, the PCR products of the normal homozygous animals (GG) presented two fragments (230 bp and 62 bp), the heterozygous animals (GA) presented three fragments (292 bp, 230 bp and 62 bp), and the animals homozygous for the mutation related to DM (AA) presented a single 292 bp fragment (Figure-1). The RFLP was validated by direct sequencing, identifying the genotype of the animals by analyzing their electropherograms (Figure-2).

The frequency of the G allele in the samples from Uruguay was 0.77, whereas that in Paraguay was 0.85. In relation to allele A, which is associated with a higher risk of DM, the frequencies were 0.15 and 0.23 for Paraguay and Uruguay, respectively (Table-1) [9, 13–20]. The observed and expected genotypic frequencies (assuming Hardy-Weinberg equilibrium) are shown in Table-2. The population of Paraguay was found in Hardy–Weinberg equilibrium (p = 1.00), whereas in dogs from Uruguay, the population deviated from equilibrium (p = 0.008). When comparing the populations, no significant difference was observed in the distribution of genotypes (p = 0.26).

**Table-1:** Allelic frequency of the SOD1:c118 G>A mutation in German Shepherd dogs from different countries.

Country	No.	Frequency of SOD1:c118 G>A	Reference
USA	6458	0.366	[9]
UK	150	0.377	[13]
Japan	541	0.220	[14]
Romania	40	0.20	[15]
Greece	29	0.138	[16]
Brazil	96	0.121	[17]
Mexico	31	0.130	[18]
USA	83	0.331	[19]
Poland	47	0.181	[19]
Israel	20	0.175	[19]
Italy	47	0.170	[20]
Uruguay	114	0.23	This study
Paraguay	44	0.15	This study

UK=United Kingdom, USA=United States of America, SOD1=Superoxide dismutase 1

**Table-2:** Observed and expected genotypic frequencies of the c. 118G>A mutation of SOD1 gene in German Shepherd dogs from Uruguay and Paraguay.

Genotype	Paraguay		Uruguay		Total	χ² p-value
	
	Observed	Expected	Observed	Expected		
GG	32 (0.64)	32 (0.64)	73 (0.72)	68 (0.60)	105 (0.66)	0.26
GA	11 (0.26)	11 (0.26)	30 (0.25)	40 (0.35)	41 (0.26)	-
AA	1 (0.1)	1 (0.1)	11 (0.03)	6 (0.05)	12 (0.08)	-

SOD1=Superoxide dismutase 1, GG=Homozygous dominant genotype, GA=Heterozygous genotype, AA=Homozygous recessive genotype

### Clinical progression rate

Neurological examinations were performed on 11 German Shepherd dogs from Uruguay with AA genotype for the SOD1:c118 G>A mutation. The age range of the animals was 3–11 years. Owners consulted their veterinary clinicians because their animals initially presented with difficulty in ambulation and/or toe dragging, with obvious wear and bleeding of the hindlimb nails. On clinical examination, in patients with evolution of <6 months, proprioceptive ataxia of the hind limbs and paraparesis with normal patellar and withdrawal reflexes (consistent with a T3-L3 myelopathy) were observed in most cases. The patients experienced no pain during spinal palpation. Only one patient (with a clinical evolution of 1.5 years) had urinary incontinence. The patient had severe muscular atrophy of the pelvic limbs and hyporeflexia. Mentation and cranial nerve examination were normal in all patients.

The animals were divided into 2 groups, one over 10 years old and the other under 10 years old (Table-3). When considering the dogs in this work, all animals older than 10 years presented with neurological symptoms suggestive of DM. Animals under 10 years of age with symptoms were in the age range of 7.5–9 years. In two animals, it was not possible to determine the exact age; however, they were elderly animals (Table-3) [14]. When considering the data from this study in accordance with previous findings by Maki *et al*. [14], 100% of the animals older than 10 years presented signs suggestive of DM. However, only 36% of the dogs under 10 years of age showed these signs (Table-3) [14].

**Table-3:** Clinical progression rate of degenerative myelopathy in German Shepherd Dogs with the AA genotype for the mutation SOD1:c118 G>A.

Age (years)	Dogs examined			Age range		Clinical signs			Rate (%)
		
	This study	[14]	Total	This study	[14]	This study	[14]	Total	
All	11	14	25	3–11	5–15	9	9	18	72
≥10 and	3	7	10	10–11	10–15	3	7	10	100
<10 and	6	5	11	3–9	5–9	4	0	4	36
Unknown	2	2	4	Unknown	Unknown	2	2	4	100

SOD1=Superoxide dismutase 1, AA=Homozygous recessive genotype

## Discussion

This study is the first report of the presence of the SOD1:c118 G>A mutation in German Shepherd dogs from Uruguay and Paraguay and the first identification of animals with clinical symptoms related to the AA risk genotype in Uruguay. Dogs from Uruguay and Paraguay were analyzed since they are close countries between which it is possible to exchange animals as management strategies for disease control.

When considering the populations of Uruguay and Paraguay together (n = 158), the frequency of the A allele responsible for DM was 0.21. This frequency is within the range established for the breed internationally (0.12–0.38) [14–18]. When considering the populations studied separately, the frequency of the mutant allele was higher in Uruguay (0.23) than in Paraguay (0.15) (Table-1) [9, 13–20]; however, a significantly different genotypic distribution was not observed between both populations (p = 0.26) (Table-2).

The allele frequencies detected in this study for both populations were lower than those reported for the United States (0.331 and 0.366) [9, 19] and the United Kingdom (0.377) [13]. In the particular case of Uruguay, the allele frequency was very similar to that reported in Japan (0.22) [14], Poland (0.181), Israel (0.175) [19], and Italy (0.17) [20], but higher than in Greece (0.138) [16], Brazil (0.121) [17], and Mexico (0.13) [18]. In the case of Paraguay, the frequency of the A allele turned out to be one of the lowest, very similar to that reported in Mexico, Brazil, and Greece (Table-1) [9, 13–20]. It should be noted that in Paraguay, only a few animals from a single geographic region were analyzed. In contrast, in Uruguay, the number of animals was greater in all regions of the country.

The high frequency of the mutant allele responsible for DM in Uruguay can be attributed to three reasons. The first is that it is a disease of late clinical onset, and the second is that its inheritance mechanism involves incomplete penetrance. In both cases, homozygous recessive animals can be used in breeding schemes before the development of symptoms if they ever do so. In addition, as a third reason, there are no control programs for the disease in Uruguay, so the risk genotype for DM does not constitute a selection criterion.

When analyzing the rate of clinical progression, using the combined data of this study and those of Maki *et al*. [14], it was observed that 100% of individuals with a risk genotype >10 years of age presented clinical symptoms compatible with DM. This indicates that the penetrance of the disease tends to be more important in older dogs, which is consistent with the fact that this is a late-onset disease [16]. Despite this, it should be emphasized that only a clinical neurological assessment was performed (this work) or surveys of symptoms to owners [14]. The confirmatory diagnosis was not reached by histopathological examination, and other diseases were not ruled out.

The high frequency of the SOD1:c118 G>A mutation in German Sheepdog dogs from Uruguay indicates the need to implement DM control measures, such as selective mating and avoiding animals with the AA genotype. In any case, these measures must be taken with caution to avoid a reduction in the effective population number and genetic erosion as a result of increased inbreeding [21], as well as the possible emergence of new recessive defects [17].

In a longitudinal epidemiological study conducted in Japan on the Welsh breed corgi between 2017 and 2022, the impact of the application of genetic tests on the frequency of the SOD1:c118 G>A mutation was observed [22]. These authors showed that the mutant allele frequency was reduced from 14.5% (2017) to 2.9% (2022) in just 5 years without any significant increase in inbreeding. This highlights the importance of selective mating with animals from other lineages or families, not carriers of the mutation [22].

Epidemiological surveys of hereditary diseases like DM are particularly important in working breeds. The German Shepherd is frequently used as a police, military, guard, or therapy dog. Training is expensive and extensive in time. For this reason, it is important to implement disease control programs in which genotyping is of special importance to guarantee a healthy and long-lasting service life [14].

The DM genotyping test should be interpreted with caution. The late onset of symptoms, added to incomplete penetrance, indicates that animals with the AA risk genotype can develop diseases with similar and treatable symptoms in isolation or concomitantly with DM. At the onset of the disease, when symptoms are incipient, orthopedic diseases such as cruciate ligament rupture, degenerative joint disease, and hip dysplasia should be ruled out. In addition, caudal abdominal pathologies, such as perineal hernia and prostate disease. When the signs are more evident, protrusive disk herniation (Hansen type II), other pathologies that produce spinal cord compression (spondylosis, joint capsule cysts, spinal neoplasms), and even infectious processes should be considered. [2]. However, genetic testing constitutes a relevant clinical tool, as revealed by Bouché *et al*. [23], where it is observed that 75% of veterinary neurologists request genotyping to establish the presumptive diagnosis of DM, and only 43 % also request spinal magnetic resonance imaging (not always available in all countries). This indicates that the presumptive diagnosis is based mainly on history, neurological findings, and genotyping tests.

## Conclusion

This study successfully identified the presence of the SOD1:c118 G>A mutation associated with a high risk of developing degenerative myelopathy (DM) in German Shepherd dogs from Uruguay and Paraguay. The allelic frequency was significant in Uruguay (0.23), while Paraguay showed a lower but notable frequency (0.15). Deviations from Hardy–Weinberg equilibrium in the Uruguayan population indicate potential selective pressures or breeding practices contributing to allele distribution. Importantly, all dogs homozygous for the mutation aged over 10 years exhibited clinical signs of DM, underscoring the severe and progressive nature of the disease. These findings emphasize the urgent need for early genetic screening to manage and mitigate DM risks in German Shepherd populations.

The findings highlight the importance of implementing systematic genotyping programs to identify and manage the SOD1 mutation in German Shepherd populations. Expanding the study to other dog breeds and conducting longitudinal clinical studies will provide deeper insights into disease progression and early intervention strategies. Developing selective breeding programs, raising regional awareness among breeders and veterinarians, and establishing genetic counseling frameworks will play a crucial role in reducing the prevalence of degenerative myelopathy while maintaining genetic diversity.

## Authors’ Contributions

RA and SL: Designed the study, collected the samples, performed PCR and RFLP assays, analyzed the data, and wrote the manuscript. AM: Designed the study and discussed the manuscript. LC, NM, and JR: Collected the samples, extracted DNA, and discussed the manuscript. CM, YP, FB, MB, and SS: Collected the samples, extracted DNA, performed PCR assays, and discussed the manuscript. All authors have read and approved the final manuscript.
